# Healthcare resource use and costs reduction with aripiprazole once-monthly in schizophrenia: AMBITION, a real-world study

**DOI:** 10.3389/fpsyt.2023.1207307

**Published:** 2023-08-04

**Authors:** Vanessa Sanchez-Gistau, María José Moreno, Susana Gómez-Lus, Antoni Sicras-Mainar, Benedicto Crespo-Facorro

**Affiliations:** ^1^Early Intervention in Psychosis Service, Hospital Universitari Institut Pere Mata, IISPV-CERCA, CIBERSAM, ISCIII, Universitat Rovira i Virgili (URV), Reus, Spain; ^2^Medical Department, Otsuka-Lundbeck Alliance, Barcelona, Spain; ^3^Medical Department, Lundbeck Alliance, Barcelona, Spain; ^4^Health Economics and Outcomes Research Department, Atrys Health, Barcelona, Spain; ^5^Department of Psychiatry, University Hospital Virgen del Rocio, IBiS, CSIC, CIBERSAM, ISCIII, School of Medicine, University of Sevilla, Sevilla, Spain

**Keywords:** schizophrenia, hospitalization, aripiprazole ILP, antipsychotic agents, healthcare resources, persistence

## Abstract

**Objective:**

This study aims to compare the hospitalization rate in individuals with schizophrenia who started their treatment with aripiprazole once monthly (AOM400) or atypical oral antipsychotics (OA) in Spain.

**Methods:**

This is an observational and retrospective study based on the electronic medical records from the BIG-PAC database. The study population consisted of individuals diagnosed with schizophrenia who initiated their treatment with AOM400 (AOM cohort) or atypical OA (OA cohort) from 01/01/2017 to 31/12/2019. A 1:1 propensity score matching (PSM) procedure was conducted to match individuals of both cohorts. The number and duration of hospitalizations, persistence to treatment, healthcare resources use, and costs were analyzed after 12 months.

**Results:**

After the PSM, 1,017 individuals were included in each cohort [age: 41.4 years (SD: 10.6); males: 54.6%]. During the follow-up period, the AOM cohort had a 40% lower risk of hospitalization than the OA group [HR: 0.60 (95% confidence interval, CI: 0.49–0.74)]. The median time to the first hospitalization was longer in individuals with AOM400 compared to those with OA (197 days compared to 174 days; *p* < 0.004), whereas hospital admissions were shorter (AOM400: 6 compared to OA: 11 days; *p* < 0.001). After 12 months, individuals receiving AOM400 were more persistent than those with OA (64.9% compared to 53.7%; *p* < 0.001). The OA cohort required more healthcare resources, mainly visits to primary care physicians, specialists, and emergency rooms than those receiving AOM400 (*p* ≤ 0.005 in all comparisons). AOM400 reduced the costs of hospitalizations, and emergency room, specialist and primary care visits by 50.4, 36.7, 16.1, and 10.9%, respectively, in comparison to the treatment with atypical OA. AOM400 led to annual cost savings of €1,717.9 per individual, from the societal perspective.

**Conclusion:**

Aripiprazole once monthly reduces the number and duration of hospitalizations, together with the treatment costs of schizophrenia, as it reduces the use of healthcare resources and productivity losses in these individuals.

## Introduction

Schizophrenia is a chronic mental illness in which perceptions, thoughts, mood, and behavior are significantly altered (1, 2). It is the most frequent psychotic disease (3), and affects approximately 1% of the global population (4), with a prevalence of 0.6% among adults in Spain (5–7). Individuals with schizophrenia have higher mortality rates than the general population, mainly due to cardiovascular and metabolic disorders, poor eating habits, nutrient deficits (8), and infectious diseases, leading to reductions in life expectancy of between 15 and 20 years (9–11). Therefore, schizophrenia implies a large and increasing burden for patients, with considerable costs for healthcare public systems and society, even in the early stages of the disease (11–15). Management costs in patients with schizophrenia with relapses almost double the costs in patients without, mainly due to the costs of hospital stays (16). In Europe, it is estimated that 44% of global total management costs of schizophrenia are indirect costs, due to productivity loss of patients and caregivers (17). In Spain, indirect costs were estimated as 71.39% of the total economic burden during the first year after the first-episode of psychosis (13).

The treatment of schizophrenia is based on antipsychotics in combination with psychotherapy and social support (1, 18–20). A person-centered treatment plan should be developed and patients have to be monitored for treatment effectiveness and side effects (20). In this sense, adherence to antipsychotics is critical for the management of this disease (21). Poor adherence is estimated at around 70% and is associated with an increased risk of relapse, longer time to remission and suicide (22, 23). Some causes of low adherence are lack of illness insight, substance abuse, negative attitude to medication, cognitive impairment and poor therapeutic alliance (21, 24–26). Lack of treatment adherence in schizophrenia, and consequent disease relapses, may also contribute to considerable healthcare resources use, with higher healthcare and indirect costs (27).

Long-acting injectable antipsychotics (LAIs) have demonstrated an improvement in treatment adherence in comparison to oral formulations (28–30). They showed better effectiveness and tolerability profile than oral antipsychotics (OA), leading to a reduction of relapses/recurrences, rehospitalizations and an improvement of some functional capacities (31–33). Therefore, the use of LAIs reduced the use of healthcare resources and improved treatment adherence/persistence with the consequent decrease in direct costs (34, 35). In this sense, a recent mirror study by Ostuzzi et al. (36) showed a decrease in hospital admissions and hospitalization days in patients persistent to LAIs. Aripiprazole once monthly (AOM400) was approved for maintenance treatment of schizophrenia in adult patients stabilized with oral aripiprazole (37). AOM400 delayed time to relapse and reduced healthcare resources in comparison to oral standard-of-care therapy (34, 38–41). A randomized head-to-head study concluded that AOM400 had superior improvements on health-related quality of life and a favorable tolerability profile, leading to a higher effectiveness, than paliperidone palmitate once-monthly (42); however, an observational prospective study using real-world data has shown no differences in effectiveness between these two drugs (43, 44). Correll et al. (45) reviewed several publications dealing with healthcare costs, some studies reported similar total costs in LAIs and OA treated-patients, while other described a decrease in treatment-related costs in LAIs-treated patients. Outpatient and pharmacy costs were usually higher in LAI-treated patients, in contrast, inpatients costs were lower, and the switch from OA to LAIs also decreased hospitalization costs. Lastly, one study modeling costs associated to LAIs or OA treatment found that LAIs were associated with lowered costs. Altogether, more evidence is needed about the consequences of the use of AOM400 in comparison to atypical OA, in terms of relapse prevention and healthcare resource utilization in clinical practice. Due to the considerable economic burden of schizophrenia, real-world information about the management costs of the disease is needed. Therefore, the AMBITION study (the Effect of Aripiprazole once - Monthly on healthcare resources use: a study Based in SpanIsh real clinical pracTIce ON schizophrenia) aims to compare in real-life conditions the incidence of hospital admissions and healthcare direct and indirect costs in individuals with schizophrenia who started their treatment with AOM400 or atypical OA in Spain, based on electronic medical records (EMRs). This will give valuable information to psychiatrists and decision makers related with AOM400 results compared to OA in routine clinical practice follow-up.

## Materials and methods

### Design

This is an observational, retrospective, cross-sectional cohort study, based on the EMRs from the BIG-PAC^®^ database. Since 2012, BIG-PAC^®^ periodically collects primary healthcare data from 1.9 million individuals who have at least one contact in primary care centers and hospitals. BIG-PAC^®^ is registered at the European Network of Centers for Pharmacoepidemiology and Pharmacovigilance, which belongs to the European Medicines Agency (46). EMRs are rigorously anonymized at the source centers, according to the Organic Law 3/2018, 5th December, on Data Protection and Guarantee of Digital Rights (47). This study was approved by the Ethics Committee of the Hospital of Terrassa (Barcelona). The patient consent was not necessary, according to the Article 5 of Royal Decree 957/2020, of November 3rd, which regulates observational studies with medicines for human use.

### Study population

The study included individuals diagnosed with schizophrenia starting a new treatment with AOM400 or atypical OA from 01/01/2017 to 31/12/2019. The start of the treatment was defined as the index date, and patients were followed up to 1 year. Individuals that initiated treatment with AOM400 (AOM cohort) were matched with the group of individuals that initiated treatment with atypical OA (olanzapine, risperidone, paliperidone, aripiprazole and asenapine – OA cohort).

Individual’s inclusion criteria were: age ≥ 18 years and ≤ 65 years; having a primary diagnosis of schizophrenia according to the International Classification of Diseases, Ninth Revision, Clinical Modification (ICD-9-CM) (code 295, including codes 295.5, 295.6, 295.8, and 295.9); being active in the database at least 12 months after the index date; being in the chronic program for prescriptions (≥2 prescriptions during the follow-up period); treatment initiation with AOM400 or atypical OA (olanzapine, risperidone, paliperidone, aripiprazole, and asenapine), and having regular monitoring, with at least 2 health records in the computer system, and sufficient data on hospitalizations during the follow-up period. Individuals who concomitantly received 2 oral atypical antipsychotics were also included in the study for the OA cohort. Concomitant treatments with oral antipsychotics were not allowed in people treated with AOM400.

Exclusion criteria were being primarily treated with typical antipsychotics, having a concomitant treatment with 2 LAIs at the index date and during the follow-up, being previously treated with clozapine, being institutionalized or terminal, and being pregnant during the study period.

### Study variables

#### Sociodemographic and clinical characteristics

The sociodemographic characteristics of the study population, age and sex, were analyzed. Clinical characteristics included time from diagnosis, the number of comorbidities and chronic diagnoses, and the estimation of the Charlson comorbidity index (48, 49), which was used as an approximation to severity. In addition, the mortality rate of the study population during the follow-up period was estimated.

#### Antipsychotic treatments

Treatments were analyzed through drug dispensing records. Drugs were prescribed to individuals according to medical practice and were identified by using the Anatomical Therapeutic Chemical Classification System (50). The study considered drugs in the N05A group, mainly oral formulations of olanzapine (N05AH03), risperidone (N05AX08), paliperidone (N05AX13), asenapine (N05AH05), and aripiprazole (N05AX12). The injectable form of aripiprazole was also considered.

The study analyzed the time from diagnosis to the first antipsychotic prescription, the percentage of patients on treatment, and the number of antipsychotic therapies received during the year before the index date. The duration (in days) of the treatment dispensed at the index date (AOM400/OA) was estimated until medication interruption (≥60 days without renewing the medication), switch to another drug, or the end of the follow-up period (1 year), whichever occurred first. In addition, the treatment continuation (persistence) was calculated as the percentage of individuals on treatment 12 months after the index date.

#### Clinical effectiveness

The clinical effectiveness of treatments was evaluated based on the annual psychiatric hospitalization rate in each cohort of the study. The definition of psychiatric hospitalization includes 2 related events: overnight stays in crisis stabilization units and/or overnight stays in psychiatric emergency departments.

The percentage of individuals in each cohort and the average number of hospitalizations per individual registered in the 12 months before and after the index date were calculated. The risk of hospitalization between AOM400 and OA cohorts was also calculated (hazard ratio, HR), as well as the time from the index date to the first hospitalization during the follow-up period.

### Resource use and costs

The use of healthcare resources during the follow-up period was estimated. Healthcare resources included: primary care and medical specialized care visits (psychiatry, psychology, and internal medicine), schizophrenia-related emergency room visits, laboratory tests, radiographies, computed tomography scans, nuclear magnetic resonance, electroencephalograms, hospitalizations, and drug treatment.

Costs were expressed as mean direct healthcare costs, indirect costs, and total costs per individual (€, 2020). Direct healthcare costs were estimated according to the use of the healthcare resources by unitary cost (Supplementary Table 1). Drug costs were calculated based on the retail price per pack at the time of prescription (51). Indirect costs included those associated with lost productivity (days of sick leave due to temporary or permanent disability in the working population) and were estimated by using the mean salary in Spain (52) (Supplementary Table 1).

### Statistical analysis

The search criteria in the database were based on computer statements (SQL scripts). Data were reviewed through exploratory analysis, searching for recording or coding errors.

A 1:1 propensity score matching (PSM) procedure was conducted to minimize confounding variables and to improve the comparability of the cohorts. Each individual in the AOM400 cohort was matched with an individual in the OA cohort. The procedure was developed according to the greedy nearest neighbor algorithm, with replacement (substitution) and accepting a caliper (tolerance) of 0.20. Exact matches were prioritized (randomly). The evaluation of the homogeneity of the cohorts was carried out using a logistic regression model and the standardized coefficients were estimated. The results of the PSM procedure were adjusted by age, sex, Charlson comorbidity index and time from diagnosis.

Descriptive univariate analyses were conducted, and qualitative data were described by using absolute and relative frequencies. Means and standard deviations [SD] were used to analyze quantitative variables with symmetric distributions, whereas medians and interquartile ranges (IQR) were used for quantitative variables with asymmetric distributions. In addition, 95% confidence intervals (CI) were calculated to estimate population parameters. The normality of distributions was checked with the Kolmogorov–Smirnov test.

Bivariate analyses (ANOVA and chi-square tests) were carried out to compare the characteristics of individuals and the effectiveness of both treatments. An analysis of covariance (ANCOVA; generalized linear model; estimate of marginal means; Bonferroni adjustment) was used to adjust costs regarding individuals’ age and sex, and Charlson comorbidity index. SPSSWIN version 23 was used, establishing a statistical significance for values of *p* < 0.05 (95% CI).

## Results

### Study population

**FIGURE 1:**
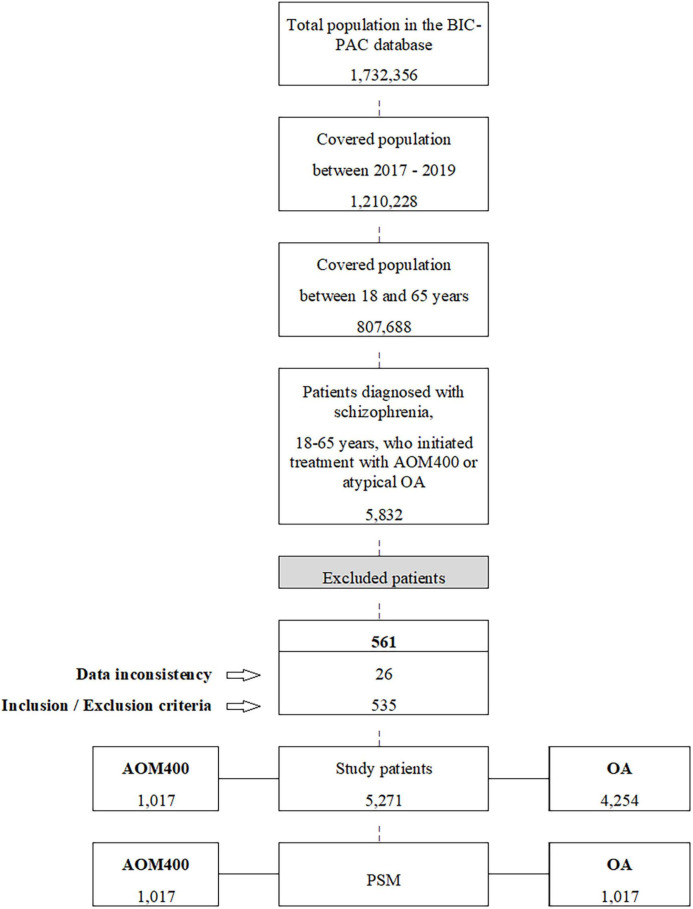
Study patients flow diagram. AOM400, aripiprazole once-monthly 400 mg; PSM, propensity score matching; OA, oral antipsychotics.

Out of a population of 1.2 million individuals who were registered between 2017 and 2019, 5,832 individuals met the inclusion criteria, as they were diagnosed with schizophrenia and had started a new treatment with AOM400 or atypical OA. Considering the exclusion criteria, 5,271 individuals were finally included in the study (1,017 and 4,254 individuals had started their treatment with AOM400 and OA, respectively) (Figure 1). The characteristics of the study population can be observed in the Supplementary Table 2.

The propensity score matching (PSM) yielded 1,017 individuals in each study cohort with similar baseline characteristics (Table 1). It was estimated that 90.6% of individuals received antipsychotic treatments (mean: 1.6 drugs [SD: 0.9]) before the index date.

**TABLE 1:** Baseline characteristics of the study cohorts after propensity score matching.

Study groups	AOM400	OA	TOTAL	*p*	SC
**Number of patients**	***N* = 1,017**	***N* = 1,017**	***N* = 2,034**		
**Sociodemographic characteristics**					
Age, years, mean (SD)	41.3 (10.8)	41.4 (10.5)	41.4 (10.6)	0.717	0.009
Males, *n* (%)	557 (54.8)	554 (54.5)	1,111 (54.6)	0.894	0.003
Time since schizophrenia diagnoses, years, mean (SD)	2.7 (1.7)	2.8 (1.8)	2.7 (1.8)	0.788	0.057
Patients who received antipsychotic therapy before the index date, *n* (%)*	928 (91.2)	915 (90.0)	1,843 (90.6)	0.323	0.022
Previous antipsychotic therapies, mean* (SD)	1.6 (0.9)	1.5 (0.9)	1.6 (0.9)	0.397	0.111
**General comorbidity**					
Chronic diseases, mean (SD)	0.9 (1)	0.8 (1)	0.8 (1)	0.103	0.100
Charlson comorbidity index, mean (SD)	0.2 (0.4)	0.2 (0.4)	0.2 (0.4)	0.999	0.006
Charlson comorbidity index, n (%)**					
0	877 (86.2)	879 (86.4)	1,756 (86.3)	0.958	0.006
1	130 (12.8)	127 (12.5)	257 (12.6)	0.839	0.003
2 +	10 (1)	11 (1.1)	21 (1)	0.825	0.005
**Types of comorbidities, n (%)**					
Arterial hypertension	198 (19.5)	167 (16.4)	365 (17.9)	0.073	0.040
Diabetes	120 (11.8)	118 (11.6)	238 (11.7)	0.890	0.003
Dyslipidemia	240 (23.6)	230 (22.6)	470 (23.1)	0.599	0.012
Obesity	171 (16.8)	172 (16.9)	343 (16.9)	0.953	0.001
Ischemic cardiopathy	8 (0.8)	2 (0.2)	10 (0.5)	0.057	0.042
Stroke	10 (1)	9 (0.9)	19 (0.9)	0.818	0.005
Heart failure	11 (1.1)	6 (0.6)	17 (0.8)	0.223	0.027
Renal failure	17 (1.7)	12 (1.2)	29 (1.4)	0.350	0.021
Depressive syndrome	101 (9.9)	90 (8.8)	191 (9.4)	0.403	0.019
Malignant neoplasms	10 (1)	6 (0.6)	16 (0.8)	0.315	0.022
Osteoporosis	25 (2.5)	27 (2.7)	52 (2.6)	0.779	0.006
Parkinson disease	10 (1)	11 (1.1)	21 (1)	0.826	0.005
Dementias (all types)	20 (2.0)	14 (1.4)	34 (1.7)	0.299	0.023
**Substance use, n (%)**					
Active tobacco smokers	252 (24.8)	290 (28.5)	542 (26.6)	0.057	0.042
Alcohol	277 (27.2)	306 (30.1)	583 (28.7)	0.155	0.032
Cocaine	79 (7.8)	100 (9.8)	179 (8.8)	0.100	0.036
Cannabis	135 (13.3)	165 (16.2)	297 (14.6)	0.038	0.046
Heroin	64 (6.3)	87 (8.6)	151 (7.4)	0.052	0.043
**Number of substances, n (%)**					
0	463 (45.5)	448 (44.1)	911 (44.8)	0.525	0.138
1	386 (38)	360 (35.4)	746 (36.7)	0.223	0.029
2	152 (14.9)	135 (13.3)	287 (14.1)	0.509	0.16
3 +	16 (1.6)	74 (7.3)	90 (4.4)	0.011	0.14

Values expressed as a percentage or mean (SD), *p*: statistical significance. * During 1 year before the index date. ** Charlson comorbidity index: a method for determining a patient’s burden of disease. AOM400, aripiprazole once-monthly 400 mg; SC, PSM standardized mean differences; OA, oral antipsychotics; SD, standard deviation.

Individuals had an average of 0.8 comorbidities (SD: 1.0), and the average Charlson comorbidity index date was 0.2 (SD: 0.4). The most frequent comorbidities were dyslipidemia (23.1%), arterial hypertension (17.9%), obesity (16.9%), and diabetes (11.7%). Most patients (61.6%) consumed at least one addictive substance, mostly alcohol (28.7%), tobacco (26.6%), and cannabis (14.6%) (Table 1).

### Treatment patterns during the follow-up

After the index date, individuals in the OA cohort were on treatment with paliperidone (40.3%), followed by aripiprazole (22.2%), risperidone (19.1%), olanzapine (16.3%), or asenapine (2.1%).

**FIGURE 2:**
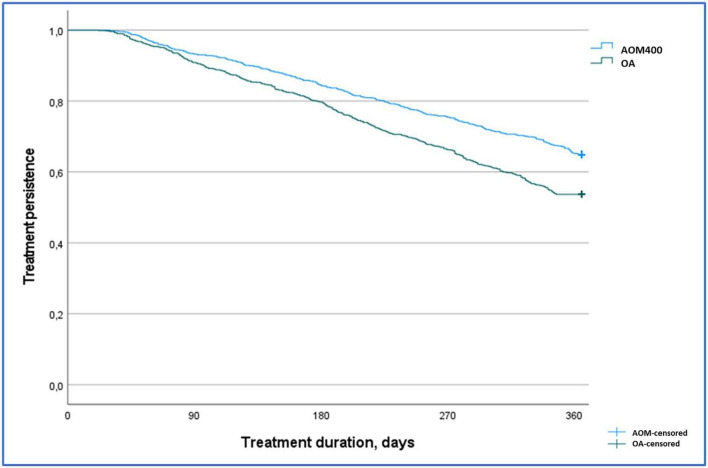
Kaplan Meier treatment persistence curve by study groups. Persistent patients after 12 months of follow-up AOM400: 64.9% vs. OA: 53.7%, *p* < 0.001. Log rank test, Chi-Squared: 27.9. AOM400, aripiprazole once-monthly 400 mg; OA, oral antipsychotics.

After 12 months of follow-up, individuals who started their treatment with AOM400 were more persistent than those that initiated treatment with atypical OA (64.9% compared to 53.7%, *p* < 0.001) (Figure 2 and Table 2). The most frequent causes of discontinuation were the interruption of treatment (23.5%), and switches to other drugs (17.3%) (Table 2).

**TABLE 2:** Persistence to antipsychotic treatment.

Study groups	AOM400	OA	Total	*p*
**Number of patients**	***N* = 1,017**	***N* = 1,017**	***N* = 2,034**	
**Treatment duration, days**				
Mean (SD)	307.3 (97.7)	285.1 (107.1)	296.2 (103.1)	<0.001
**Persistence to treatment**				
N° of patients persistent after 12 months, *n* (%)	660 (64.9)	546 (53.7)	1,206 (59.3)	<0.001
Discontinuation, *n* (%)	357 (35.1)	471 (46.3)	828 (40.7)	<0.001
-Switch to other drugs	150 (14.7)	201 (19.8)	351 (17.3)	<0.001
-Interruption	207 (20.4)	270 (26.5)	477 (23.5)	0.001
Death, *n* (%)	8 (0.8)	3 (0.3)	11 (0.5)	0.131

Values expressed as a percentage or mean (SD), *p*: statistical significance. The treatment duration was estimated as the period from the index date to medication interruption (≥60 days without renewing the medication), switch to another drug, or the end of the study (until 31/12/2020), whichever occurred first. AOM400, aripiprazole once-monthly 400 mg; OA, oral antipsychotics; P, percentile; SD, standard deviation.

### Use of healthcare resources

#### Psychiatric hospitalizations

**FIGURE 3:**
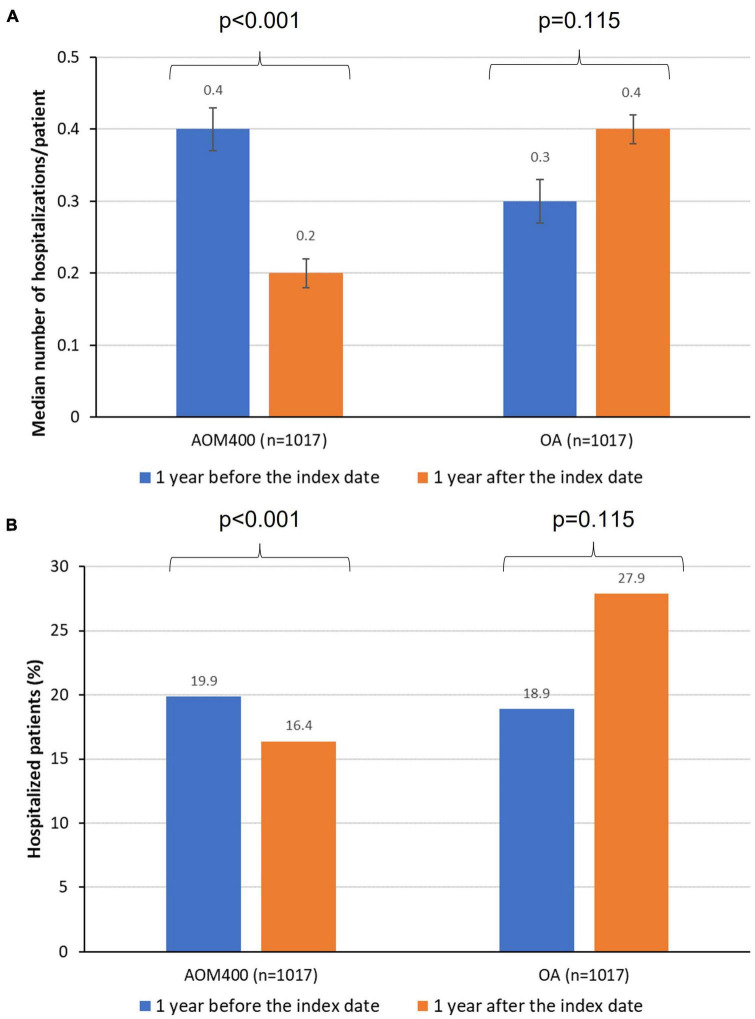
Number of hospitalizations per patient **(A)**, and hospitalized patients **(B)** in both periods of the study. AOM400, aripiprazole once-monthly 400 mg; OA, oral antipsychotics.

**FIGURE 4:**
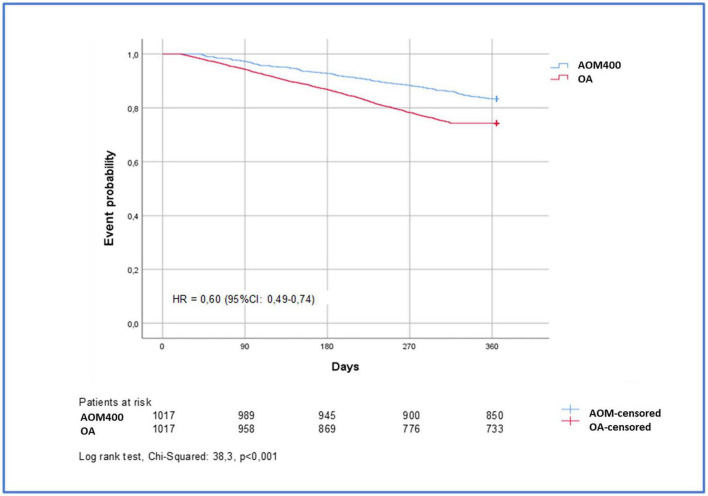
Time to first psychiatric hospitalization after initiation with AOM400 or OA. AOM400, aripiprazole once-monthly 400 mg; OA, oral antipsychotics; HR, hazard ratio; CI, confidence interval.

In the year before the index date, there were no differences in the number of individuals hospitalized nor the average number of psychiatric hospitalizations per individual in the study cohorts. However, during the follow-up period, psychiatric hospitalizations were more frequent in the OA cohort than in the AOM cohort (27.9% compared to 16.4%, *p* < 0.001). The OA cohort doubled the average hospitalization rate per individual in comparison to individuals in the AOM cohort (0.4 compared to 0.2, *p* < 0.001) (Figure 3 and Table 3). Therefore, the AOM cohort had a 40% lower risk of psychiatric hospitalizations than the OA cohort [HR: 0.60 (95% CI: 0.49–0.74)] (Figure 4). It should be noted that the OA cohort had fewer hospitalizations during the year before vs. after the index date, whereas individuals with AOM400 showed a reduction in the psychiatric hospitalizations rate in those study periods (before the index date: 19.9% compared to after the index date: 16.4%) (Figure 3 and Table 3).

**TABLE 3:** Hospitalizations per patient before and after the index date.

Study groups	AOM400	OA	Total	*p*
**Number of patients**	***N* = 1,017**	***N* = 1,017**	***N* = 2,034**	
**Hospitalizations during the year before the index date**				
Hospitalized patients due to psychiatric causes, *n* (%)	202 (19.9)	192 (18.9)	394 (19.4)	0.575
Number of hospitalizations due to psychiatric causes per patient, mean (SD)	0.4 (0.9)	0.3 (0.8)	0.4 (0.9)	0.115
**Hospitalizations during the follow-up**				
Hospitalized patients, *n* (%)	167 (16.4)	284 (27.9)	451 (22.1)	<0.001
N° of hospitalizations per patient, mean (SD)	0.2 (0.6)	0.4 (0.7)	0.3 (0.7)	<0.001
**Number of hospitalizations per patient, n (%)**				
0	850 (83.6)	733 (72.1)	1,583 (77.8)	<0.001
1	101 (9.9)	174 (17.1)	275 (13.5)	<0.001
2	55 (5.4)	94 (9.2)	149 (7.3)	0.001
3 +	11 (1.1)	16 (1.6)	27 (1.3)	0.329
**Time to first hospitalization, days**				
Mean (SD)	197.9 (95.9)	173.1 (82.3)	182.3 (88.3)	0.004
Median (P25-P75)	197 (105–285)	174 (101–245)	183 (103–257)	
**Duration of hospitalizations, days**				
Mean	4.6 (12.3)	9.4 (16.9)	7.0 (15)	<0.001
Median	6 (3–12)	11 (8–18)	9 (7–15)	

Values expressed as mean (SD) per patient/year, *p*: statistical significance. The follow-up period was 1 year from the index date (the start of the AOM400/OA treatment). AOM400: aripiprazole once-monthly 400 mg; OA: oral antipsychotics; SD: standard deviation.

The median time to the first psychiatric hospitalization was longer in individuals treated with AOM400 compared to those with atypical OA (197.9 days compared to 173.1 days; *p* < 0.004); in addition, the median duration of hospital admissions was longer in individuals treated with atypical OA in comparison to AOM400 (11 compared to 6 days; *p* < 0.001) (Table 3).

#### Other healthcare resources

Individuals that initiated treatment with OA required more visits to primary care, specialized care, and emergency rooms than those receiving AOM400 (*p* ≤ 0.005 in all comparisons, Table 4). The OA cohort also required more sick leave days than the AOM400 cohort (17.9 compared to 10.2 days; *p* < 0.001) (Table 4).

**TABLE 4:** Use of healthcare resources and costs per patient during the follow-up.

Study groups	AOM400	OA	Total	*p*
**Number of patients**	***N* = 1,017**	***N* = 1,017**	***N* = 2,034**	
**Use of healthcare resources**				
Primary care visits	10.0 (8.9)	11.2 (10.7)	10.6 (9.9)	0.005
Specialized visits	14.5 (14.6)	17.3 (14.9)	15.9 (14.8)	<0.001
Emergency rooms visits	0.2 (0.5)	0.3 (0.5)	0.3 (0.5)	<0.001
Laboratory tests	1.1 (0.5)	1.1 (0.4)	1.1 (0.5)	0.029
Radiographies	0.3 (0.5)	0.2 (0.4)	0.3 (0.4)	0.004
Computed tomography scans	0 (0.2)	0.1 (0.2)	0 (0.2)	0.173
Nuclear magnetic resonances	0 (0.1)	0 (0.1)	0 (0.1)	0.592
Electroencephalograms	0.5 (1)	0.5 (0.9)	0.5 (1)	0.981
Hospitalizations, *n* (%)	167 (16.4)	284 (27.9)	451 (22.1)	<0.001
Duration of hospitalizations, mean, days (SD)	4.6 (12.3)	9.4 (16.9)	7.0 (15)	<0.001
Duration of sick leaves, mean, days (SD)	10.2 (14.9)	17.9 (34.3)	14 (26.7)	<0.001
**Costs, mean (SD), €**				
Primary care visits	230.8 (207.2)	259.1 (247.6)	244.9 (228.7)	0.005
Specialized visits	1,335 (1,343.8)	1,592.1 (1,369)	1,463.6 (1,362.2)	<0.001
Emergency rooms visits	25.7 (54.4)	40.6 (63.2)	33.1 (59.4)	<0.001
Laboratory tests	24.7 (10.8)	23.7 (9.9)	24.2 (10.4)	0.029
Radiographies	5.2 (8.3)	4.2 (7.8)	4.7 (8.1)	0.004
Computed tomography scans	3.8 (18.7)	5 (21.8)	4.4 (20.3)	0.173
Nuclear magnetic resonance	1.4 (15.6)	1 (13.6)	1.2 (14.6)	0.592
Electroencephalogram	20.4 (36.7)	20.3 (33.8)	20.3 (35.3)	0.981
Hospitalizations	1,955.5 (5,197.4)	3,946.2 (7,092.4)	2,950.9 (6,295.2)	<0.001
Antipsychotic drugs	2,568 (1,663.3)	1,267.6 (872.5)	1,917.8 (1,478.5)	<0.001
**Direct healthcare costs, €**	**6,170.5 (5,603.6)**	**7,159.9 (7,457.8)**	**6,665.2 (6,613.1)**	0.001
**Indirect costs (sick leave days), €**	**1,035.5 (1,508.6)**	**1,806.6 (3,471.1)**	**1,421 (2,703.2)**	<0.001
**Total costs, €**	**7,206 (6,447.9)**	**8,966.4 (9,824.7)**	**8,086.2 (8,354.1)**	<0.001
Adjusted costs (ANCOVA)*			**Difference**	***p***
**Direct healthcare costs**				
Mean	6223.7	7173.9	−950.2	0.001
CI 95%	5817.6–6629.8	6768.0–7579.8		
**Indirect costs**				
Mean	1033.3	1801.0	−767.7	<0.001
CI95%	868.5–1198.1	1636.3–1965.8		
**Total costs**				
Mean	7257.0	8974.9	−1717.9	<0.001
CI95%	6745.5–7768.5	8463.8–9486.1		

Values expressed as mean (SD) per patient/year, *p*: statistical significance. The follow-up period was 1 year from the index date (the start of the AOM400/OA treatment). *ANCOVA analysis adjusted for: age, sex, and Charlson index. The bold values indicate the total direct (primary care visits+specialised visits, etc.,) and indirect costs and the overall total costs (the sum of direct and indirect costs). ANCOVA, analysis of covariance; AOM400, aripiprazole once-monthly; CI, confidence interval; OA, oral antipsychotics; SD, standard deviation.

### Costs

Individuals treated with AOM400 had lower direct healthcare costs than individuals treated with atypical OA [€6,170.5 (SD: 5,603.6) compared to €7,159.9 (SD: 7,457.8)]. The most relevant cost categories were antipsychotic treatments, hospitalizations, and specialized visits. AOM400 increased pharmacological costs by €1,300.4 but reduced the costs of hospitalizations (−€1,990.7; 50.4%), specialist visits (−€257.1; 16.1%), primary care visits (−€28.3; 10.9%), and emergency room visits (−€14.9; 36.7%) in individuals with schizophrenia (Table 4).

Adjusted healthcare costs amounted to €6,223.7 [95% CI: 5817.6–6629.8] and €7,173.9 [95% CI: 6768.0–7579.8], in the AOM400 and the OA cohorts, respectively, leading to a reduction of −€950.2. Indirect costs were also higher in individuals treated with atypical OA compared to those with AOM400 [€1,801.0 (95% CI: 1,636.3–1,965.8) compared to €1,033.3 (95% CI: 868.5–1,198.1)]. Therefore, the use of AOM400 in individuals with schizophrenia offset the pharmaceutical costs, leading to annual cost savings of €1,717.9 per individual, from the societal perspective (Table 4).

Regarding indirect costs, in terms of productivity losses, we estimated an average of 14.0 workdays lost per year per patient (Table 4). Of note, our results show a reduction in the number of sick leave days in patients treated with AOM400 10.2 (SD, 14.9) as compared to OA 17.9 days (SD, 34.3) (*p* < 0.001)].

## Discussion

Schizophrenia represents substantial costs for healthcare systems, particularly due to the considerable number of hospitalizations, which constitutes the largest component of healthcare costs (15). To our knowledge, this is the largest study analyzing the use of healthcare resources and treatment costs with AOM400 and atypical OA in a real-world setting in Europe. Our study showed that individuals who started their treatment with AOM400 had a 40% lower risk of hospitalization than those who initiated their therapy with atypical OA after the first year of treatment. We observed a reduction in the average number of hospitalizations per individual by 50.0% in the AOM400 cohort when comparing the year before and after the index date. Other studies reported even higher reductions in the hospitalization rates associated to AOM400 (34, 38). Wilson et al. (34) reported a 63.8% reduction in hospitalizations in the 6 months before and after AOM400 treatment initiation, whereas Potempa and Rychlik (38) observed reductions of 74.6% in the 6 months after the switch to aripiprazole-depot.

We estimated that hospitalizations in patients with schizophrenia lasted an average of 7.0 days, which according to the authors is a conservative estimation, although a previous study reported shorter hospital stays, with a median length of 4.0 days (5). The differences may derive from the fact that the study by Orrico-Sanchez et al. (5) considered all therapeutic alternatives for schizophrenia (including first and second generation oral and injectable antipsychotics), whereas we only considered AOM400 as LAI. In addition, our results showed that individuals who started the AOM400 treatment had a longer time to hospitalization and shorter hospitalizations than those with atypical OA. We estimated that AOM400 reduced the length of hospital admissions by 51.1% during the follow-up period, in comparison to the OA cohort. In this sense, Wilson et al. (34) reported a reduction in the length of hospital admissions of 76.1%, in the 6 months before and after AOM400 treatment initiation, whereas Potempa et al. (38) observed 79.6% reductions in the length of hospitalizations.

The treatment persistence during the first year was also higher in the AOM400 cohort compared to the OA cohort (64.9% compared to 53.7%; *p* < 0.001). Similar results were reported in the PROSIGO and DOMINO trials, which analyzed treatment persistence after starting AOM400 treatment in the real-world setting in Spain and Italy, respectively. They observed that the patients on treatment with AOM400 had a persistence of 71.4 and 86.0% after 6 months of the start of the treatment (53). Additionally, another study carried out in Germany reported that AOM patients were more persistent to the treatment than those receiving atypical OA (40.7 and 19.8%, respectively) after 1 year of treatment (54). These improvements may be associated to the once-monthly long-acting injectable formulation and the superiority of AOM400 compared to OA in terms of effectiveness and tolerability (31–33). They may be associated with a reduction in the incidence of relapses, with the consequent decrease in the use of healthcare resources and costs. The randomized clinical trial reported by Naber et al. (42) showed improvements in the tolerability profile and HR-QoL through the validated Heinrichs–Carpenter Quality-of-Life Scale, which is in contrast with the results obtained in the prospective study of Bartoli et al. (43, 44). Differences in the methodology employed in these two studies may have determined the comparable effectiveness in LAIs and OA found by Bartoli et al. (43, 44). Our results have an observational origin, with real-world data from hospitals and primary care centers, and are in line with results obtained in the abovementioned studies. Nevertheless, other studies have not shown differences between LAIs or OA treatments. In this sense, a systematic review and meta-analysis highlighted that published evidence on LAIs or OA efficacy and tolerability was not conclusive (55). As previously suggested, these differences may point to the specific formulation or administration characteristics of these compounds, which have been shown to play a role in their rate of absorption and/or side effects; additionally, drug interactions causing wrong dosage could also have a negative effect (56). Moreover, a recently published randomized clinical trial (the EULAST study), which focused on time to all-cause discontinuation, concluded that no significant advantage of LAIs over OAs was observed regarding their capacity to avoid discontinuation in early phase schizophrenia patients (57). A comment to this study highlighted the number of publications reporting benefits of LAIs in different aspects, and that the EULAST study was the first to report comparable benefits of LAIs and OA, suggesting that these results may have been due to the methodology employed (58). Noteworthy, the follow-up carried out in the EULAST study differs from the one frequently performed in the clinical practice and could have influenced the results with a potential positive impact in adherence.

Regarding the use of other healthcare resources for the treatment of schizophrenia, our study population had an average of 10.6 and 15.9 visits to primary care centers and specialists, respectively. These results are higher than the ones estimated by other regional study carried out in Spain (5), in which the study population had a median of 3.8 and 0.96 visits per person-year to primary care and specialists, respectively. We estimated that during the first year of the treatment, the primary care and specialist visits were reduced by 10.7 and 16.2%, respectively, whereas Potempa et al. (38) observed even higher reductions in the number of psychiatric visits, of 27.4%.

In terms of productivity losses, we estimated an average of 14.0 days, whereas Orrico-Sánchez et al. (5) estimated that the median sick leave days were 12.5 days, respectively. The variations may be associated with differences in the study population, as they considered patients from a specific region in Spain, whereas we included individuals from a larger database that is representative of our country. Nevertheless, our results showed that the use of AOM400 reduced the sick leave days by 43.0%, compared to OA.

The reduction in the use of healthcare resources leads to decreases in the management costs of schizophrenia. Our study showed that the AOM cohort had a reduction in direct and total healthcare costs, 13.2% (−€950.2) and 19.1% (−€1,717.9), respectively, compared to the OA cohort. In line with our results, Wilson et al. (34) reported a 7.1% (−€1,046.0) reduction with the use of AOM400 in direct healthcare costs during a follow-up period of 6 months. However, Potempa et al. (38) estimated that AOM400 could reduce a 53.2% (−€5,048.5) the direct healthcare costs per individual during the first 6 months of therapy. In addition to the differences in the management of schizophrenia, the variations may be related to the cost components, since Wilson et al. (34) only considered the antipsychotic treatment and hospitalization costs, whereas Potempa et al. (38) included pharmacological treatment, hospitalizations, day hospital care, and medical and emergency visits (34, 38). Therefore, our results evidenced that the use of AOM400 reduced the use of healthcare resources, such as the number and length of hospital admissions and medical visits (primary care centers, specialists, and emergency rooms), in comparison to atypical OA. Hence, the administration of AOM400 offsets its pharmacological costs, being a cost-effective treatment for individuals with schizophrenia (59, 60).

Our study also has some limitations. First, BIG-PAC is an administrative database with limitations when used for observational studies; it may have incomplete patient follow-up data, particularly if individuals changed to centers outside the area of influence. Second, the possible classification bias of individuals and disease categorization may affect the quantification of healthcare resource use and costs. Third, due to the characteristics of the database, it was not possible to estimate direct non-healthcare costs, such as those associated with formal or informal care. Fourth, the study did not consider variables that may influence the results such as individuals’ socioeconomic status. Fifth, the study focused on the use of healthcare resources and costs in the first year of the AOM or OA treatments, but these results may vary in the subsequent years. Sixth, due to the study design, treatment duration was only estimated in the first year after the index date. Seventh, our study showed that the use of substances (particularly tobacco) was lower than previous data in individuals with schizophrenia (61–63). These differences may be associated with the under-registration of this variable in electronic medical records.

## Conclusion

The use of AOM400 for the treatment of schizophrenia reduced the use of healthcare resources and productivity losses in comparison to atypical OA, particularly in terms of the number and the duration of hospitalizations. Our real-world results evidenced that AOM400 offsets its pharmacological costs, mainly due to the reduction of hospitalizations and medical visit costs in Spain. From the societal perspective, the treatment with AOM400 led to cost savings of €1,717.9 per individual in the first year of treatment.

## Data availability statement

The original contributions presented in the study are included in the article/Supplementary material, further inquiries can be directed to the corresponding author.

## Ethics statement

The studies involving human participants were reviewed and approved by Comitè d’Ètica d’Investigació Clínica del Consorci Sanitari de Terrassa. Written informed consent for participation was not required for this study in accordance with the national legislation and the institutional requirements.

## Author contributions

AS-M, SG-L, and MM, conceived the study. AS-M, SG-L, MM, BC-F, and VS-G participated and contributed to the study design. AS-M made data collection and the statistical analysis. All authors contributed to the interpretation of the data and critically reviewed and approved the final version of the manuscript.
