# Supplementary material for: Healthcare resource use and costs reduction with aripiprazole once-monthly in schizophrenia: AMBITION, a real-world study
Source: Front Psychiatry. 2023 Aug 4;14:1207307. doi: 10.3389/fpsyt.2023.1207307 (PMC10437073; doi:10.3389/fpsyt.2023.1207307)
Supplement: Supplementary file 1 [file Table_1.docx]

Supplementary Material

Table S1. Unitary costs (year 2020)

| **Healthcare and non-healthcare resources** | **Unitary cost (€)** |
| --- | --- |
| *Medical visits* |  |
| Medical visits to primary care | 23.19 |
| Medical visits to the emergency room | 117.53 |
| Hospitalization (per day) | 420.90 |
| Medical visit to specialized care* | 92.00 |
| *Supplementary tests* |  |
| Lab tests | 22.30 |
| Conventional radiology | 18.50 |
| Axial computed tomography | 96.00 |
| Magnetic nuclear resonance | 177.00 |
| Diagnostic test: electroencephalogram | 37.12 |
| *Pharmaceutical prescription* | RP VAT |
| *Laboral productivity - indirect costs*** |  |
| Cost per not worked day | 101.20 |

* Considered: psychiatry, psychology, and internal medicine.

** Estimation of the average earnings by age and sex in the Spanish active population.

RP: retail price.

Table S2. Baseline characteristics of the study population before propensity score matching.

| **Study groups** | **AOM400** | **OA** | **TOTAL** | **p** |
| --- | --- | --- | --- | --- |
| Number of patients (%) | N=1017 (19.3) | N=4254 (80.7) | N=5271 |  |
| *Sociodemographic characteristics* |  |  |  |  |
| Age, years; mean (SD) | 41.3 (10.8) | 44.0 (11.2) | 43.5 (11.2) | <0.001 |
| Males, n (%) | 557 (54.8) | 2392 (56.2) | 2949 (56.0) | 0.481 |
| Patients who received antipsychotic therapies before index date*, n (%) | 928 (91.2) | 3750 (88.2) | 4679 (88.8) | 0.005 |
| Previous antipsychotic treatments*, mean (SD) | 1.6 (0.9) | 1.5 (0.9) | 1.5 (0.9) | 0.100 |
| Hospitalized patients due to psychiatric causes, n (%) | 202 (19.9) | 799 (18.) | 1001(18.9) | 0.42 |
| Nº of hospitalizations due to psychiatric causes per patient, mean (SD) | 60.4 (0.9) | 0.3 (0.8) | 0.3 (0.8) | 0.05 |
| *General comorbidity* |  |  |  |  |
| Chronic diseases, mean (SD) | 0.9 (1) | 0.8 (1.1) | 0.8 (1.1) | 0.707 |
| Charlson comorbidity index, mean (SD) | 0.2 (0.4) | 0.2 (0.5) | 0.2 (0.5) | 0.275 |
| Charlson comorbidity index, n (%)** |  |  |  |  |
| 0 | 880 (86.3) | 3713 (87) | 4593 (86.8) | 0.004 |
| 1 | 130 (12.7) | 441 (10.3) | 571 (10.8) | 0.027 |
| 2+ | 10 (1) | 115 (2.7) | 125 (2.4) | 0.001 |
| *Types of comorbidities, n (%)* |  |  |  |  |
| Arterial hypertension | 198 (19.5) | 790 (18.6) | 986 (18.7) | 0.616 |
| Diabetes | 120 (11.8) | 442 (10.4) | 560 (10.6) | 0.264 |
| Dyslipidemia | 240 (23.6) | 1031 (24.2) | 1271 (24.1) | 0.658 |
| Obesity | 171 (16.8) | 662 (15.6) | 833 (15.8) | 0.332 |
| Ischemic cardiopathy | 8 (0.8) | 30 (0.7) | 38 (0.7) | 0.785 |
| Stroke | 10 (1) | 31 (0.7) | 41 (0.8) | 0.408 |
| Heart failure | 11 (1.1) | 49 (1.2) | 60 (1.1) | 0.847 |
| Renal failure | 17 (1.7) | 55 (1.3) | 72 (1.4) | 0.352 |
| Depressive syndrome | 101 (9.9) | 305 (7.2) | 406 (7.7) | 0.003 |
| Malignant neoplasms | 10 (1.0) | 41 (1.0) | 51 (1.0) | 0.957 |
| Osteoporosis | 25 (2.5) | 110 (2.6) | 135 (2.6) | 0.814 |
| Parkinson disease | 10 (1.0) | 55 (1.3) | 65 (1.2) | 0.420 |
| Dementias (all types) | 20 (2.0) | 88 (2.1) | 106 (2.0) | 0.540 |
| *Consumption of substances, n (%)* |  |  |  |  |
| Active smokers | 252 (24.8) | 1182 (27.8) | 1434 (27.2) | 0.051 |
| Alcohol | 277 (27.2) | 1205 (28.3) | 1480 (28.1) | 0.403 |
| Cocaine | 79 (7.8) | 398 (9.4) | 476 (9.0) | 0.090 |
| Cannabis | 135 (13.3) | 751 (17.7) | 886 (16.8) | 0.001 |
| Heroin | 64 (6.3) | 374 (8.8) | 438 (8.3) | 0.009 |
| *Number of substances, n (%)* |  |  |  |  |
| 0 | 463 (45.5) | 1777 (36.3) | 2206 (41.9) | <0.001 |
| 1 | 386 (38) | 1549 (36.4) | 1959 (37.2) | <0.001 |
| 2 | 152 (14.9) | 588 (13.8) | 745 (14.1) | 0.764 |
| 3+ | 16 (1.6) | 355 (8.3) | 379 (7.2) | 0.001 |

Values expressed as a percentage or mean (SD), p: statistical significance.

* During 1 year before the index date. ** Charlson comorbidity index: a method for determining a patient’s burden of disease.

AOM400: aripiprazole once-monthly 400 mg;; OA: oral antipsychotics; SD: standard deviation.
